# Enhancing the Interfacial Adhesion of a Ductile Gold Electrode with PDMS Using an Interlocking Structure for Applications in Temperature Sensors

**DOI:** 10.3390/nano15131001

**Published:** 2025-06-28

**Authors:** Shuai Shi, Penghao Zhao, Pan Yang, Le Zhao, Jingguang Yi, Zuohui Wang, Shihui Yu

**Affiliations:** School of Power and Electrical Engineering, Luoyang Institute of Science and Technology, Luoyang 471023, China; 18800703861@163.com (S.S.); dlzg_92@163.com (P.Y.);

**Keywords:** embedded structure, interfacial adhesion, interlocking effect, temperature sensor, Au/PDMS

## Abstract

The poor interfacial adhesion between ductile gold (Au) electrodes and polydimethylsiloxane (PDMS) substrates affects their application in flexible sensors. Here, a porous Au electrode is designed and combined with a flexible PDMS substrate to form a structure that embeds Au into the PDMS film, thereby enhancing the interfacial adhesion of the Au/PDMS electrode. The resistivity change of the Au/PDMS electrode is only 12.3% after 100 tape peeling trials. The resistance of the Au/PDMS electrode remains stable at the 30% strain level after 2000 tensile cycling tests. This feature is mainly attributed to the deformation buffering effect of the porous Au film. After 100 min of ultrasonic oscillation testing, the resistivity change of the Au/PDMS electrode remains stable. It is also shown that the Au/PDMS electrode has excellent interfacial adhesion properties, which is mainly attributed to the interlocking effect of the Au/PDMS electrode structure. In addition, the temperature coefficient of resistance (TCR) of the temperature sensor based on the Au/PDMS electrode is approximately 0.00320/°C and the sensor’s sensitivity remains almost stable after 200 temperature measurement cycles. Au/PDMS electrodes have great potential for a wide range of applications in flexible electronics due to their excellent interfacial adhesion and electrical stability.

## 1. Introduction

In recent years, flexible temperature sensors have found extensive application in personal medical care and disease diagnosis owing to their remarkable sensitivity, rapid response time, and excellent repeatability [1,2,3,4,5,6,7,8]. Flexible and stretchable electrodes, as a key component of these sensors, provide an important platform for studying the electrophysiological characteristics of tissues in vivo and in vitro, as well as for treatment [9,10]. These electrodes are typically composed of organic elastic polymers and conductive materials. Due to the significant differences in the physical and chemical properties between these two materials, their interfacial adhesion is usually insufficient, especially for metal films on elastic substrates [11]. This weak adhesion can cause the conductive layer to fall off, thereby reducing the durability and efficiency of electronic devices [12]. Especially when the electrode needs to have mechanical flexibility and tensile strength, this weak adhesion is even more fatal.

The solution to this problem entails developing an electrode that not only exhibits strong interfacial adhesion but is also both flexible and stretchable. PDMS is extensively employed in the fields of electronics and biomedicine owing to its superior viscoelastic properties, chemical and mechanical stability, low cost, and ease of fabrication [13,14]. Considering the superior properties of PDMS, its integration with various conductive materials as an organic elastic substrate has been extensively studied [15,16,17,18,19,20]. These studies primarily aim to enhance the interfacial adhesion between the conductive layer and the substrate via physical interactions, chemical crosslinking, and topological adhesion. For instance, conductive materials such as metals, carbon-based materials, and conductive polymers have been directly embedded into PDMS to form elastic conductive composites [21,22,23]. Nanoparticle Au electrodes have been combined with cured PDMS to form a mutually embedded structure, which improves the adhesion between the conductive layer and the elastic substrate [11]. In addition, the elastic substrate can be treated with oxygen plasma to promote the physical interaction between the conductive layer and the newly formed hydroxyl groups on the substrate surface, thereby enhancing the interfacial adhesion [24]. However, the preparation process of chemical cross-linking limits its application due to its excessive complexity.

A wide variety of composite electrodes have been fabricated by combining flexible and stretchable conductive materials with PDMS substrates [25,26,27,28,29,30,31]. Metal materials have become the preferred conductive layer due to their excellent electrical conductivity and excellent stability [32,33]. In particular, metals exhibit flexibility when sufficiently thin and can be engineered into specific geometries that enable folding or stretching [34,35]. A composite electrode was prepared by depositing a Au layer on PDMS, but the deposited Au nanoparticles (AuNPs) were prone to falling off due to the poor compatibility of AuNPs with PDMS [36]. Furthermore, AuNPs were sputtered onto the pre-stretched PDMS surface by magnetron sputtering to prepare AuNP–PDMS composite films. However, the physical adsorption effect between AuNPs and PDMS is relatively weak, and AuNPs are prone to falling off [37]. PDMS colloid was uniformly mixed with a chloroauric acid solution at a temperature of 70 °C and continually heated until PDMS was completely cured, synthesizing spongy AuNP–PDMS composites. However, the preparation method is rather complex, and the conductivity of Au is weakened [38]. In a typical case, a metallic nanopile array was prepared by means of electrochemical methods using alumina (AAO) films as templates [39,40]. Then, the PDMS solution was poured into the metal nanocolumn array and solidified to form a chimeric structure [11]. This structure does enhance the binding force between the metal nanoarray and PDMS, but the electrochemical preparation method is complex in process and too costly.

Based on the above considerations, in this study, nanoporous Au is prepared by means of the dealloying method as the conductive layer material, and Au/PDMS electrodes are fabricated in combination with PDMS flexible substrates. The Au film exhibits a thickness of only 100 nm and it has a bicontinuous ligament and pore structure. The adhesion of the electrode interface is significantly enhanced by embedding Au films into the PDMS substrate. After 100 stripping tests, the resistance change rate of the Au/PDMS electrode is 12.3%. Furthermore, the resistance of the Au/PDMS electrode maintains stability in bending–recovery fatigue tests and tension–recovery fatigue tests. In addition, the flexible stretchable temperature sensor prepared based on these electrodes has strong sensitivity and stability, enabling accurate temperature monitoring in the range of 30 °C to 80 °C.

## 2. Materials and Methods

The preparation process of Au was as follows [41]: initially, a 12 K Au-Ag alloy film with a thickness of 100 nm was subjected to corrosion in a 65 wt.% HNO_3_ solution for 2 h at 40 °C to produce the Au film. Subsequently, the Au was thoroughly rinsed three times with ultrapure water to eliminate any residual nitric acid and was then stored in ultrapure water for subsequent use. The preparation process of Au/PDMS electrodes and sensors is elaborated in detail later.

The morphological characteristics of the samples were examined using field emission scanning electron microscopy (FE-SEM, JSM-6700F, Shōjima City, Japan). The bending-cycle and tension-cycle tests were conducted utilizing a small flexible tensile testing system (FlexTest-Mini-S-P2, Hunan Nasheng Electronic Technology Co., Ltd. Changsha, China). Electrical resistance measurements at varying temperatures were performed with an inductive capacitance resistance analyzer (HIOKI IM3523, Ueda City, Nagano Prefecture, Japan). The temperature sensing performance was evaluated using an oil bath heating method (Shanghai Lichen Co., Ltd., Shanghai, China), which offers temperature control accuracy of ±0.1 °C within a testing range of 30 to 160 °C.

## 3. Results and Discussion

**Figure 1:**
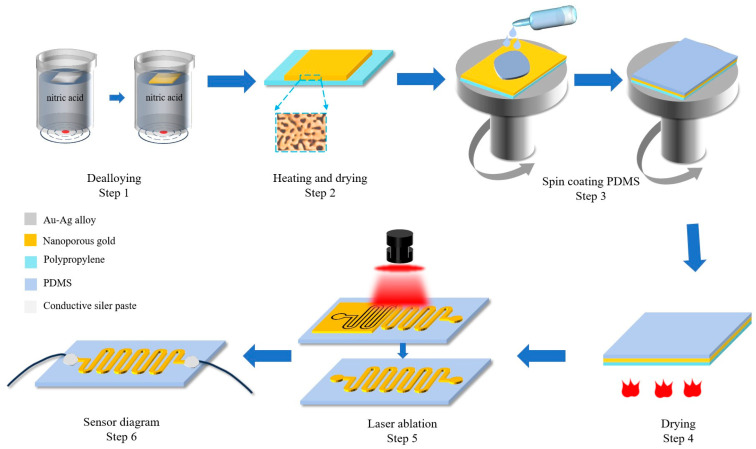
Schematic of the fabrication procedure of Au/PDMS temperature sensors.

The preparation process of the Au/PDMS temperature sensor is illustrated in Figure 1. Initially, an alloy film composed of gold and silver was transferred to a nitric acid solution on a slide for etching purposes, thereby removing the majority of the silver content and yielding a Au film with distinctive nanoporous characteristics. The total thickness of this film measured only 100 nm. Following the etching of silver, the Au content in the Au films ranged from 50 to 100 μg/cm^2^, remaining within an economically viable range. Subsequently, the Au film was transferred onto a polypropylene substrate and dried at 60 °C for 30 min. Following this, an appropriate amount of PDMS solution was spin-coated on the Au surface at 500 rpm for 50 s. The PDMS was then evenly distributed using spin coating technology and subsequently dried. Finally, by peeling the Au/PDMS film off the polypropylene substrate, a flexible and stretchable Au/PDMS electrode was obtained. Then, under the conditions of a voltage of 220 V, a power of 40 W, a laser wavelength of 355 nm, and an accuracy of 0.1 mm, the electrode was laser-etched to form a serpentine pattern with a width of 2 mm (Step 5), thereby preparing the final Au/PDMS temperature sensor [42]. There were no obvious Au debris residues in the laser-etched area. As illustrated in Step 6 of Figure 1, the pattern design of the temperature sensor features a size of 20 mm × 20 mm, with a line width and gap of 2 mm each. The resistance-sensitive part consists of a Au conducting layer embedded in the PDMS film, which has excellent electrical conductivity and interface adhesion.

**Figure 2:**
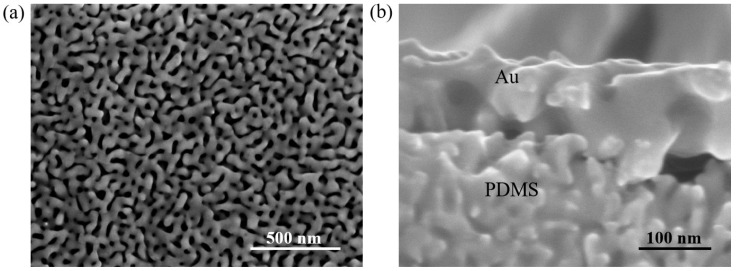
(**a**) FE-SEM images of Au with the Au embedded into the PDMS films; (**b**) cross-sectional FE-SEM micrograph of the Au/PDMS temperature sensor.

Figure 2 illustrates the morphology and structure of the Au/PDMS electrode. Figure 2a depicts the morphological characteristics of the Au conductive layer on the surface of the Au/PDMS electrode. The average pore size of the Au layer is about 50 nm and it has a unique bicontinuous structure consisting of pore channels and ligaments [41]. Compared with traditional dense metal films, this porous structure endows the metal films with higher flexibility. This is because the porous structure enables the metal ligaments between the holes of the film to bend and deform relatively easily when subjected to force, thereby adapting to the action of external forces and avoiding cracking or breaking as easily as dense films. Figure 2b shows the cross-sectional morphology of the Au/PDMS electrode. As can be seen from Figure 2b, the PDMS is partially immersed in the Au film, which indicates that the structure of this Au film is conducive to the permeation of low-molecular-weight PDMS molecules. When the PDMS solution is coated on the Au surface, part of the PDMS solution will penetrate into the Au channel. After a curing process, a structure is formed in which the Au is embedded in the PDMS film. This embedded configuration generates a mutual interlocking effect, significantly enhancing the adhesion between the Au conductive layer and the PDMS substrate. This structural design mimics the natural mechanism by which tree roots anchor soil firmly, thereby improving the adhesion at the Au/PDMS electrode interface.

**Figure 3:**
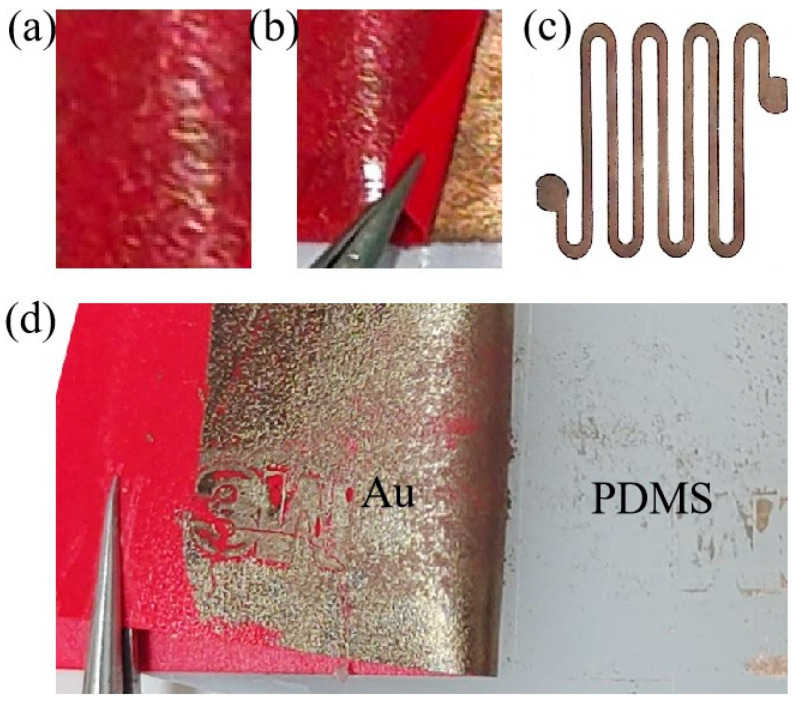
(**a**) Three-layer composite film of tape/Au/PDMS; (**b**) tape/Au/PDMS peeling-off test; (**c**) temperature sensor based on the Au/PDMS electrode; (**d**) tape/Au-PDMS peeling-off test.

Figure 3 shows the process diagram of the Au/PDMS electrode peeling experiment. Figure 3a shows three-layer composite film of tape/Au/PDMS. Figure 3b visually describes the process of repeatedly peeling off the Au/PDMS electrode with 3M tape. Here, it should be noted that the porous Au film in the electrode is embedded in the PDMS substrate. The lower the Au content on the 3M tape after peeling, the greater the bonding strength between Au and the base. As shown in Figure 3b, after peeling off the red 3M tape from the Au surface, there are no fine traces of Au fragments on the tape. These intuitive results indicate that the interfacial adhesion of the Au/PDMS embedded electrode is much greater than that between the Au and the tape. This is mainly because the interface in the Au/PDMS electrode has a mechanical binding force with a chain effect. As shown in Figure 3d, a porous Au film was placed on a PDMS membrane and dried to form an Au/PDMS electrode. After peeling the tape off the Au surface, almost all the Au electrodes adhered to the tape. This was mainly because there was only van der Waals force between the Au film and the PDMS membrane in this electrode. Finally, a temperature sensor was fabricated based on the Au/PDMS electrode (Figure 3c).

**Figure 4:**
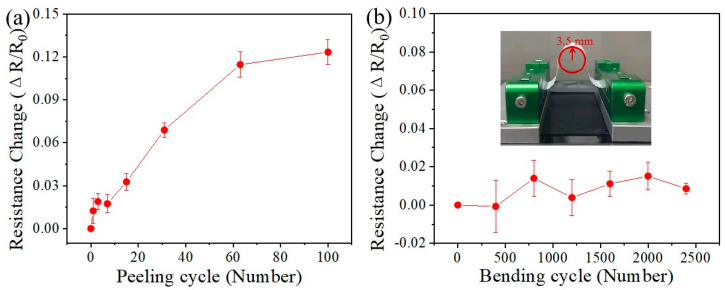
(**a**) Resistance change of the Au/PDMS electrode as a function of peeling-off cycle; (**b**) resistance change of the Au/PDMS electrode as a function of the bending cycle at a bending radius of 3.5 mm.

Figure 4a also illustrates the relationship between the resistance change of the Au/PDMS electrode and the number of stripping cycles. The initial resistance of Au is 4.6 Ω, with a corresponding electrical conductivity of 2.2 × 10^6^ S·m^−1^. This value is in good agreement with previous studies [11,41]. After 100 stripping tests, the resistance change rate of the Au/PDMS electrode was only 12.3%. These test results verify the interface structure shown in Figure 3b. The combination between the porous Au electrode and the PDMS substrate forms a nested structure. This physical structure has an interlocking effect, which greatly enhances the interfacial adhesion of the electrode.

To further validate the high adhesion at the interface of the Au/PDMS electrode, a bending–recovery experiment was performed. The change rate of resistance during the bending process is calculated as follows [43]:

(1) $$R_{s}=\frac{\Delta R}{R_{0}}=\frac{\left(R_{c}-R_{0}\right)}{R_{0}}$$

where R_s_ denotes the change rate of resistance, ΔR represents the alteration in resistance following a bending cycle, while R_0_ and R_c_ correspond to the initial resistance and the measured resistance after the bending cycle. As illustrated in Figure 4b, under a fixed bending radius of 3.5 mm and a relative strain of approximately 17.5%, the resistance of the Au/PDMS electrode is evaluated across multiple bending–recovery cycles. As shown in Figure 4b, the experimental data show that after 2400 bending–recovery tests, the resistance of the Au/PDMS electrode is essentially unchanged (<2%). This suggests that the electrode’s electrical performance is highly stable. Such remarkable stability can be attributed primarily to the interlocking embedded structure between the Au conductive layer and the PDMS substrate, which significantly enhances interfacial adhesion and helps to maintain their original electrical properties under considerable bending stress.

**Figure 5:**
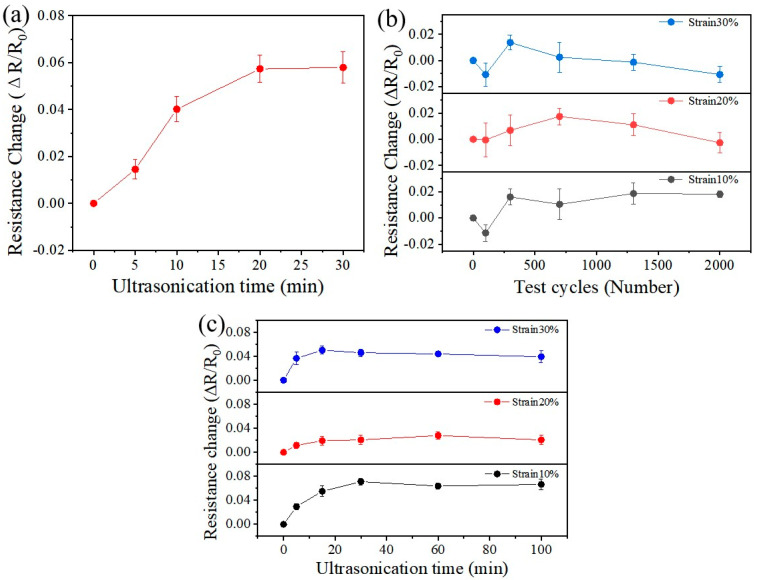
(**a**) Resistance changes of Au/PDMS electrodes as a function of ultrasonic cleaning time; (**b**) resistance changes of Au/PDMS electrodes after stretching cycle; (**c**) resistance changes of Au/PDMS electrodes after alternating stretch recovery and ultrasonic cleaning.

Figure 5a shows the variation in the resistance of the Au/PDMS electrode with its ultrasonic oscillation cleaning time. It can be clearly seen that as the ultrasonic oscillation time increases, the resistance of the electrode increases, but the increase amplitude is relatively small. After ultrasonic cleaning for 20 min, the mean resistance value of the Au/PDMS electrode is 12.84 Ω and the resistance change rate is stably at 5.7% compared with the initial resistance. The experimental results demonstrate the high stability of the interface structure of the Au/PDMS electrode, thereby maintaining stable electrical performance. Subsequently, the tensile recovery properties of Au/PDMS electrodes were tested at different strain levels (10–30%). These electrodes must withstand continuous mechanical stress because they need to conform to the dynamic stress of human skin [43]. As shown in Figure 5b, at the 10% strain level, the resistance change rate of the Au/PDMS electrode was less than 2% after 2000 cycles. The research test was extended to the 20% and 30% strain levels. After cycling 2000 times at these higher strain levels, the electrode resistance values did not change significantly either. The stability of the electrode resistance is mainly due to the deformation buffering effect of the porous gold electrode. The three-dimensional porous structure of the porous gold electrode deforms like a spring under the action of an external force. The concentrated stress is dispersed through the pore network and transformed into uniform deformation. Within a certain strain range, the gold skeleton remains continuous, thereby maintaining the stability of its resistance. These tensile test results show that the embedded structure of the electrode effectively improves the tensile performance of the electrode and increases the strain tensile range of the electrode. This enables the electrode to be applied in a wider range of scenarios and maintain stable electrical performance.

The resistance changes of the Au/PDMS electrode were evaluated after alternating stretching and recovery and ultrasonic cleaning. As illustrated in Figure 5c, when the electrode is subjected to a 10% strain, it undergoes 20 initial stretch–recovery cycles, followed by 5 min of ultrasonic cleaning, and the corresponding resistance is recorded. Subsequently, the electrode undergoes another 20 stretch–recovery cycles, followed by 15 min of ultrasonic cleaning, with the resistance measured again. Upon repeating this process multiple times, the resistance of the Au/PDMS electrode remains stable (from 20 to 100 min). The resistance change rate of the Au/PDMS electrode under 20% and 30% tensile strain was evaluated. These test results indicate that the resistance value of the Au/PDMS electrode has not changed significantly either (from 20 to 100 min). In Figure 5, three destructive tests were conducted on the Au/PDMS electrode. The experimental results fully demonstrate that the Au/PDMS electrode has a high interfacial adhesion and excellent structural stability, further verifying the experimental results in Figure 2 and Figure 3.

**Figure 6:**
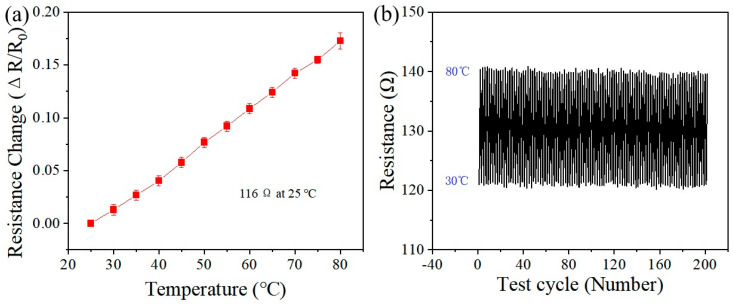
(**a**) Resistance variation of Au/PDMS temperature sensors at 5 °C intervals; (**b**) resistance variation of Au/PDMS temperature sensors in the temperature range from 30 °C to 80 °C as a function of heating and cooling cycle.

The experimental results above demonstrate that the Au/PDMS electrode structure exhibits superior stability and consistent electrical properties. A Au/PDMS temperature sensor with a serpentine bending structure was successfully fabricated by precisely controlling the size of Au in the Au/PDMS electrode (Figure 6a). At 25 °C, the resistance value of the Au/PDMS temperature sensor is 116 Ω. Figure 6a presents the characteristic curve of the resistance of the Au/PDMS temperature sensor as a function of temperature. The experimental findings indicate that due to Au having a positive TCR, the rate of resistance of the sensor increases significantly with rising temperature. The resistance exhibits a strong linear relationship with temperature, suggesting that the TCR value of the sensor remains constant and is unaffected by temperature variations. The formula for calculating TCR is provided as follows [44,45,46]:

(2) $$TCR=\frac{dR}{dT}\frac{1}{R_{0}}$$

where R denotes the resistance of the sensor at temperature T and R_0_ represents the resistance at room temperature. The calculated TCR values change from 0.00312/°C to 0.00329/°C for the sensors. This is probably due to the humidity adsorption effect of the 100 nm thick porous Au film at low temperatures, which slightly reduces the TCR. When the temperature is increased, water molecules are desorbed from the surface of the porous Au film and the TCR is thus appropriately increased. The Au/PDMS temperature sensor measures resistance changes at 5 °C intervals. As the temperature increases, the sensor’s resistance exhibits significant variation, indicating that the Au/PDMS temperature sensor can accurately detect marginal temperature changes of 5 °C. Furthermore, to validate the reliability of the Au/PDMS temperature sensor, a temperature cycle test was carried out, in which the temperature varied from 30 °C to 80 °C during 200 heating and cooling cycles. As shown in Figure 6b, after 200 temperature cycles, the resistance does not change significantly at 80 °C, which is mainly due to the high stability of the Au/PDMS temperature sensor.

**Figure 7:**
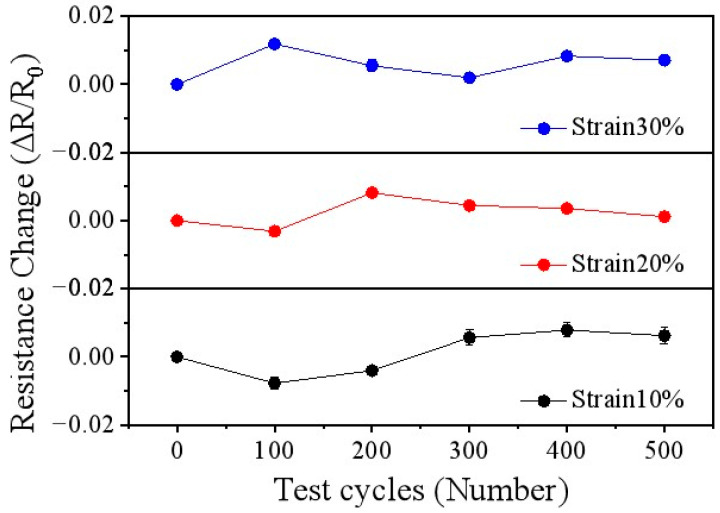
Resistance changes of Au/PDMS temperature sensor as a function of test cycles.

Figure 7 illustrates the resistance variations of the Au/PDMS temperature sensor at various tensile strain levels (10–30%) to evaluate its electrical stability during the process of application to human skin [47,48]. As depicted in Figure 7, when the strain level is set at 10%, after 500 stretching cycles, the resistance change rate of the Au/PDMS temperature sensor is less than 2% and can be within the error range in practical applications. Further tests at the 20% and 30% strain levels also showed that the resistance of the sensor also did not change significantly, which was mainly attributed to the deformation buffering effect of the porous Au electrode. When the porous Au film is subjected to tensile force, its curved part will gradually straighten out. Some originally weak connection points may gradually break, while some adjacent ligaments may approach each other due to stretching, forming new connection points. Therefore, the formation of new connection points and the disconnection of old connection points keep the electron transmission path basically stable, thereby maintaining the basic stability of the resistance.

## 4. Conclusions

In summary, a high-adhesion Au/PDMS electrode has been successfully fabricated in this study by embedding a conducting Au layer into a PDMS thin film substrate. Subsequently, a high-performance Au/PDMS temperature sensor with robust interface adhesion and superior stability is developed using laser etching technology. After 100 tape peeling tests, the resistance change rate of the Au/PDMS electrode is 12.3%. During 2000 tensile tests with a tensile strain of 10–30% and 2400 bending–recovery tests with a bending radius of 3.5 mm and a relative strain of approximately 17.5%, it is found that the resistance of Au remains essentially stable. In addition, the Au/PDMS electrode has been tested by alternating ultrasonic cleaning and tensile testing. The results show that the Au/PDMS electrode has high stability. In the temperature range from 30 °C to 80 °C, the TCR value of the Au/PDMS temperature sensor is about 0.00320/°C and shows an essentially linear response. The results of temperature cycling and tensile testing additionally confirmed the outstanding electrical stability of the Au/PDMS temperature sensor. These remarkable properties indicate that the Au/PDMS structural design provides a valuable platform for the development of flexible sensor devices in the future.

## Data Availability

All data generated or used in the study are presented in the submitted article.
